# *In vitro* evaluation of olorofim and antifungal combinations against *Aspergillus* and *Candida* species

**DOI:** 10.1093/jac/dkaf354

**Published:** 2025-09-30

**Authors:** Corinne Pinder, Ressa Lebedinec, Jason D Oliver, Mike Birch, Derek Law

**Affiliations:** F2G Ltd, Alderley Park, Macclesfield, Cheshire SK10 4TF, UK; F2G Ltd, Alderley Park, Macclesfield, Cheshire SK10 4TF, UK; F2G Ltd, Alderley Park, Macclesfield, Cheshire SK10 4TF, UK; F2G Ltd, Alderley Park, Macclesfield, Cheshire SK10 4TF, UK; F2G Ltd, Alderley Park, Macclesfield, Cheshire SK10 4TF, UK

## Abstract

**Background and Objectives:**

*Aspergillus* and *Candida* spp. are important causes of systemic fungal infections. Although various therapies are available, resistance to current antifungal treatments, particularly azoles, is increasing. Combinations of antifungals can be used to treat infections with resistant pathogens. In a recent *in vitro* study, antagonism was reported in a single *A. fumigatus* isolate between olorofim and voriconazole. Available clinical data are limited but do not reflect this phenomenon. The *in vitro* interactions of olorofim with current antifungal agents (voriconazole, posaconazole, isavuconazole, fluconazole, amphotericin B, terbinafine and caspofungin) were evaluated against various *Aspergillus* and three *Candida* species.

**Methods:**

*In vitro* interactions were evaluated by the EUCAST microdilution broth technique modified for checkerboard assay.

**Results:**

Olorofim demonstrated different interaction patterns when tested in combination with various classes of antifungal. Unidirectional antagonism was seen between olorofim and the mould-active azoles with the strongest effect seen in *A. niger*: azole MIC values were unaffected but olorofim MIC values increased, although remained within wild-type distributions. Olorofim antagonized amphotericin B activity in the single *A. niger* strain tested. There was indifference between olorofim and fluconazole, terbinafine and caspofungin for all isolates. Finally, olorofim showed indifference with fluconazole or voriconazole against three *Candida* species.

**Conclusions:**

Mould-active azoles antagonize olorofim activity against *Aspergillus* spp. combination MICs remain within wild-type distributions for *Aspergillus* spp., other than for *A. niger.* In addition, olorofim does not affect the anti-*Candida* effect of fluconazole and could be co-dosed where necessary without loss of the effect of the azole against the yeast.

## Introduction

*Aspergillus* spp. are a predominant cause of systemic fungal infections; recent estimates suggest aspergillosis affects ∼4 million people annually in chronic or invasive forms.^1^ Currently, invasive aspergillosis (IA) carries a >95% mortality rate when untreated and mortality rate that approaches 50% even if diagnosed and treated properly.^1^ While the aspergillosis burden is large and increasing,^2^ only three classes of antifungal drugs are approved for its treatment. The triazoles, which target ergosterol synthesis, are the recommended first-line agents for treating IA followed by the polyene amphotericin B,^3,4^ but resistance to the former is increasingly problematic^5–7^ and the latter has significant renal toxicity issues.^3^ Azole resistance among isolates of *A. fumigatus* is rising and is frequently linked to triazole fungicide exposure in agriculture and horticulture.^8^ Echinocandins are often used to treat *Candida* infections and are sometimes used to treat *Aspergillus* infections if azoles and polyenes are contraindicated.^3^ Terbinafine also affects the ergosterol biosynthetic pathway, has been used in combination with olorofim against bronchopulmonary *Microascus* infections^9^ and is sometimes used in combination with azoles to treat infections caused by *Scedosporium* spp. and *Lomentospora prolificans*.^10,11^

To overcome resistance and toxicity issues, the discovery and development of new classes of antifungals is required. Olorofim is a novel dihydroorotate dehydrogenase inhibitor with activity against a range of pathogenic fungi^12–14^ including some dimorphic fungi and difficult-to-treat species^15–18^ but excluding yeast^12^ and Mucorales^19^ due to variation in the nature of the target enzyme. It is highly active against *Aspergillus* species *in vitro*, including isolates with intrinsic^20^ or acquired^21^ resistance to existing antifungals. Importantly, intrinsic resistance to olorofim is negligible in clinically important *Aspergillus* spp.^22,23^ and no significant cross-resistance with azoles has been detected within these species.^21–23^ Further, development of olorofim resistance *in vitro* is relatively rare in *A. fumigatus*: the spontaneous rate of resistance was found to be relatively low, appearing to fall below the rate for itraconazole, but above the rate for voriconazole in four of five tested strains.^23^ In addition, during repeated exposures to drug, decreased susceptibility appeared earlier for voriconazole than olorofim.^12^ Olorofim recently completed a Phase II clinical trial (ClinicalTrials.gov Identifier NCT03583164) for salvage treatment of patients with aspergillosis, rare mould infections and coccidioidomycosis,^24^ and is now in a Phase III clinical trial for IA (ClinicalTrials.gov Identifier NCT05101187).

To counteract antifungal resistance or for refractory infections, combination therapy with two or more drugs is sometimes used to achieve a more potent antifungal effect^5^ but this approach is not always based on supportive *in vitro* evidence.^25^ Recent work reported an antagonistic relationship between voriconazole and olorofim against wild-type but not L98H/TR34 mutant *A. fumigatus in vitro.*^26^ However, this study employed unstandardized methodology for antifungal susceptibility testing of filamentous moulds^27^ as well as non-standard interpretations of fractional inhibitory concentration index (FICI) values.^25,28^ Moreover, this *in vitro* phenomenon may not extend to all susceptible species; olorofim and itraconazole behaved indifferently in combination against *Madurella mycetomatis*.^29^ While an increase in the olorofim minimum inhibitory concentration (MIC) was observed in combination with voriconazole for both azole-susceptible and azole-resistant *A. fumigatus*,^26^ the resultant 4-fold-increased olorofim MIC was still within wild-type susceptibility distributions^22^ and reflective of susceptibility in line with the Phase II data in which patients receiving olorofim monotherapy or in combination with an azole had similar clinical outcomes.^30^ Furthermore, a recent investigation of olorofim pharmacodynamics in combination with posaconazole *in vivo* also observed antagonism between the two agents in a murine model challenged with an azole-susceptible *A. fumigatus* strain, but found that combination therapy still achieved greater therapeutic effects than either monotherapy.^31^ This was not the case for an azole-resistant strain: only olorofim monotherapy suppressed infection compared to azole or combination treatment, consistent with a therapeutic approach that would avoid azole use in this situation.

To characterize the relationship between olorofim and other antifungals using standard methodology, a comprehensive *in vitro* combination susceptibility study was undertaken against a panel of *Aspergillus* isolates. The panel comprised standard control, clinical and laboratory strains and included two different azole-resistant *A. fumigatus Cyp51A* mutants. Olorofim was tested in combination with voriconazole, posaconazole, isavuconazole, fluconazole, amphotericin B, terbinafine and caspofungin using checkerboard European Committee for Antimicrobial Susceptibility Testing (EUCAST) microdilution broth assays, as these agents are likely candidates for combination therapy. Although olorofim has no activity against yeasts, its effect on fluconazole and voriconazole activity was tested against several species of *Candida* as combination treatment may be encountered in patients with mould/*Candida* co-infections or in patients receiving azole prophylaxis.

## Materials and methods

### Strains and culturing

All strains (Table 1) were stored as spore/cell suspensions in 10% glycerol at −80°C. *Aspergillus* species were cultured on Sabouraud dextrose agar (SAB, Oxoid) and grown at 35°C for 5 days to sporulate. *A. fumigatus*, *A. terreus*, *A. flavus* and *A. niger* were selected as the most common pathogenic *Aspergillus* species, and azole-resistant strains of *A. fumigatus* were included as these are increasingly frequent in the clinic. Among the strains tested were two recommended for quality control^33^: ATCC204305 and ATCC204304. *Candida* species were cultured on SAB at 35°C for 2 days. The quality control strain *C. parapsilosis* ATCC2019 was included,^33^ as well two of the most common pathogenic *Candida* species, *C. albicans* and *C. glabrata*, that represent fluconazole-susceptible and -resistant yeast species.^34^

**Table 1.** List of strains used in this study

Strain	Code	Information
*Aspergillus fumigatus*	AF293	Standard lab strain
*Aspergillus fumigatus*	NIH4215	ATCCMYA-1163, used in olorofim PK/PD models^32^
*Aspergillus fumigatus*	ATCC204305	EUCAST control strain ATCC204305
*Aspergillus fumigatus*	F17294	Azole-resistant L98H/TR34 mutant
*Aspergillus fumigatus*	F11628	Azole-resistant G138C mutant
*Aspergillus flavus*	ATCC204304	EUCAST control strain ATCC204304
*Aspergillus terreus*	AT4	Clinical strain
*Aspergillus niger*	AN1	Clinical strain
*Candida parapsilosis*	ATCC22019	EUCAST control strain ATCC22019
*Candida albicans*	CA	Fluconazole-susceptible lab strain
*Candida glabrata*	CG	Fluconazole-resistant lab strain

### Checkerboard assays

*In vitro* activity of the combination of olorofim with the other antifungals of interest was evaluated using the EUCAST broth microdilution ISO method for moulds^27^ or yeasts^35^ modified for a checkerboard assay to test two agents in combination. Stock solutions of olorofim (OLO, F2G Ltd.), voriconazole (VOR, Biosynth), posaconazole (POS, Biosynth), isavuconazole (ISA, Merck), fluconazole (FLU, Sigma), amphotericin B (AMP, Sigma), terbinafine (TER, Sigma) and caspofungin (CAS, Sigma), were prepared in DMSO. All experiments were performed in duplicate in Nunc 96-well plates (Thermo Scientific).

For olorofim, the azoles, amphotericin B and terbinafine, a 100% growth inhibition endpoint was reported as the MIC and for caspofungin the minimum effective concentration (MEC) was reported for the *Aspergillus* spp. As olorofim lacks activity against yeasts due to target enzyme variation^12^ and precipitates in microdilution testing conditions at ∼12.5 mg/L, determination of exact MIC values is not achievable for olorofim against *Candida* spp. Therefore, 8 mg/L olorofim was used as the maximum concentration against *Candida* spp. to prevent precipitation affecting endpoint reading and results and olorofim MIC values were recorded as off scale. For the drugs tested against *Candida* spp., a 50% growth inhibition endpoint was reported as the MIC.

All MICs and MECs were determined visually, including for *Candida* spp., which deviates from current EUCAST guidelines for approved drugs with anti-yeast activity.^35^ To be consistent with the methodology applied to reading endpoints against moulds, all *Candida* spp. MIC values were determined visually, as is accepted by the Clinical and Laboratory Standards Institute.^36^ The *Candida* spp. MIC values for azoles determined in this study fell within the EUCAST wild-type MIC distributions.^37–39^

All results were reported as the range in the tables and graphed as the geometric mean (GM) where applicable.

### Fractional inhibitory concentration index calculation

After MICs or MECs were determined for each agent alone and in combination, the FICI was calculated. An FICI ≤0.5 indicated synergy (S) between two agents, between >0.5–4 indicated indifference (I), and >4 indicated antagonism (A).^25,28^ For agents that had MIC or MEC values above the highest concentration tested, the next concentration above the highest was used in FICI calculation. Where combination MICs or MECs were less than or equal to the lowest concentration of agent tested, the lowest tested concentration was used in FICI calculation. Raw MIC or MEC values (0.01563, 0.03125) were used to calculate an accurate FICI value rather than rounded concentration values (0.016, 0.03), and the results reported as ranges.^40^

## Results

### Voriconazole antagonizes olorofim in a strain-dependent manner in Aspergillus spp.

**Figure 1.**
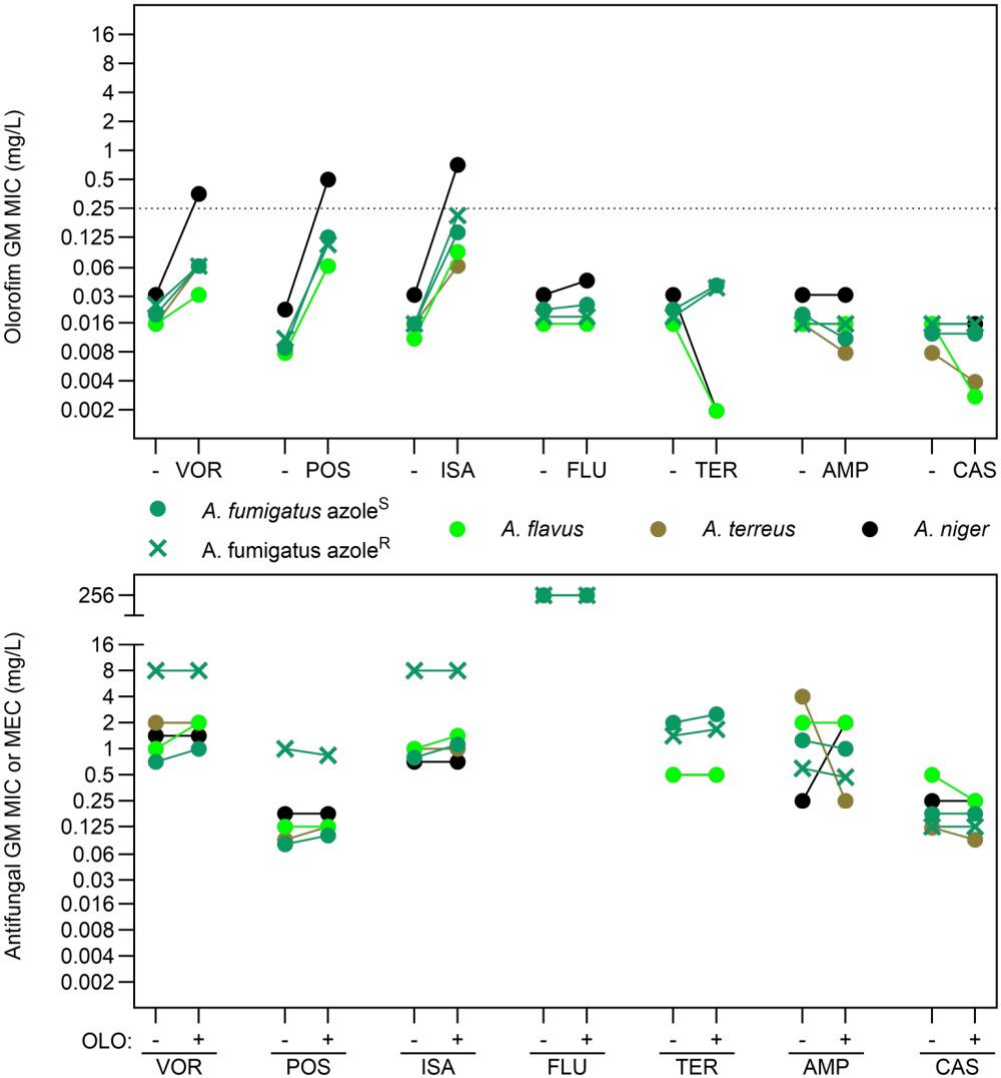
Summary of olorofim and antifungal agent MIC changes. Top, GM olorofim MIC values in the absence (−) or presence of the antifungal agents tested. Dotted line indicates the upper limit of wild-type MIC distribution for olorofim.^22^ Bottom, GM MIC or MEC values for the antifungal agents tested in the absence (−) or presence (+) of olorofim. VOR, voriconazole; POS, posaconazole; ISA, isavuconazole; FLU, fluconazole; TER, terbinafine; AMP, amphotericin B; CAS, caspofungin; OLO, olorofim. Note some datapoints may overlap.

Overall, voriconazole and olorofim interacted antagonistically (FICI 5-17) or indifferently (FICI 3 or 4) (Table 2 and Figure 1). Olorofim MICs were consistently higher by at least one (NIH4215, F17294 and ATCC204304) or two dilutions (AF293, ATCC204305, F11628 and AT4) in the presence of the azole. Antagonism was strongest in *A. niger* where voriconazole caused an 8- to 16-fold increase in olorofim MIC to 0.25 or 0.5 mg/L. Conversely, the effect of olorofim on voriconazole was negligible with single dilution increases in voriconazole MIC observed for three of the eight strains (AF293, NIH4215 and ATCC204304).

**Table 2.** Interaction between olorofim and voriconazole in *Aspergillus* spp.

	Agent alone (mg/L)		Combination (mg/L)			
Strain	OLO	VOR	OLO	VOR	FICI	Interaction
AF293	0.016	1	0.06	1–2	5–6	A
NIH4215	0.03	0.5	0.06	1	4	I
ATCC204305	0.016	0.5–1	0.06	0.5–1	5	A
F17294	0.03	4–8	0.06	4–8	3	I
F11628	0.016	16	0.06	16	5	A
ATCC204304	0.016	1	0.03	2	4	I
AT4	0.016	2	0.06	2	5	A
AN1	0.03	1–2	0.25–0.5	1–2	9–17	A

OLO, olorofim; VOR, voriconazole; A, antagonism; I, indifference.

### Posaconazole antagonizes olorofim activity in all Aspergillus spp. tested

Posaconazole antagonized olorofim activity in all isolates (FICI 8.5–32.5), raising olorofim MICs in both azole-susceptible and azole-resistant strains (Table 3 and Figure 1). The strongest antagonistic effect was seen in *A. niger* where the olorofim MIC increased 16- to 32-fold. By contrast, the effect of olorofim on posaconazole was negligible with single dilution increases or decreases in posaconazole MIC observed for five of the eight isolates (AF293, F17294, F11628, AT4 and AN1).

**Table 3.** Interaction between olorofim and posaconazole in *Aspergillus* spp.

	Agent alone (mg/L)		Combination (mg/L)			
Strain	OLO	POS	OLO	POS	FICI	Interaction
AF293	0.008	0.125	0.06–0.125	0.25	10–18	A
NIH4215	0.008	0.06	0.125	0.06	17	A
ATCC204305	0.008–0.016	0.06	0.125–0.25	0.06	17	A
F17294	0.016	0.5–1	0.125	1	9–10	A
F11628	0.008	1–2	0.06–0.125	0.5–1	8.5–16.5	A
ATCC204304	0.008	0.125	0.06	0.125	9	A
AT4	0.008	0.06–0.125	0.06	0.125	9–10	A
AN1	0.016–0.03	0.125–0.5	0.5	0.125–0.25	18–32.5	A

OLO, olorofim; POS, posaconazole; A, antagonism.

### Isavuconazole antagonizes olorofim activity in all Aspergillus spp. tested

Isavuconazole antagonized olorofim activity in all isolates tested (FICI 5–33), regardless of azole-susceptibility status (Table 4 and Figure 1). The strongest antagonism was seen in *A. niger* and the two azole-resistant *A. fumigatus* isolates, where the olorofim MIC increased 16- to 32-fold. Although isavuconazole clearly reduces olorofim activity *in vitro*, olorofim remained active against these moulds as the elevated MICs generally remained below 1 mg/L. Conversely, the effect of olorofim on isavuconazole was negligible with a single dilution increase in the isavuconazole MIC observed for only two *A. fumigatus* isolates (AF293 and ATCC204305) and for the *A. flavus* isolate (ATCC204304).

**Table 4.** Interaction between olorofim and isavuconazole in *Aspergillus* spp.

	Agent alone (mg/L)		Combination (mg/L)			
Strain	OLO	ISA	OLO	ISA	FICI	Interaction
AF293	0.016	1	0.125–0.25	2	10–18	A
NIH4215	0.016	1	0.125	1	9	A
ATCC204305	0.016	0.5	0.125	0.5–1	9–10	A
F17294	0.016	>4	0.125–0.25	>4	9–17	A
F11628	0.016	>4	0.25	>4	17	A
ATCC204304	0.008–0.016	1	0.06–0.125	1–2	9–10	A
AT4	0.016	1	0.06	1	5	A
AN1	0.03	0.5–1	0.5–1	0.5–1	17–33	A

OLO, olorofim; ISA, isavuconazole; A, antagonism.

### Fluconazole and olorofim behave indifferently against Aspergillus spp., even at high concentrations of fluconazole

The effect of high fluconazole concentrations on olorofim activity was tested against the panel of *Aspergillus* species (Table 5 and Figure 1). The fluconazole MIC was above the highest concentration tested (>128 mg/L) for all strains. Olorofim and fluconazole susceptibility remained unchanged in combination for six of the eight strains. Two isolates (AF293 and AN1) showed a single dilution increase in olorofim MIC in the presence of fluconazole; this was only observed at the highest concentration of fluconazole tested, and produced an FICI of 3, indicating indifference between the two agents.

**Table 5.** Interaction between olorofim and fluconazole in *Aspergillus* spp.

	Agent alone (mg/L)		Combination (mg/L)			
Strain	OLO	FLU	OLO	FLU	FICI	Interaction
AF293	0.016	>128	0.016–0.03	>128	2–3	I
NIH4215	0.03	>128	0.03	>128	2	I
ATCC204305	0.016–0.03	>128	0.016–0.03	>128	2	I
F17294	0.016–0.03	>128	0.016–0.03	>128	2	I
F11628	0.016	>128	0.016	>128	2	I
ATCC204304	0.016	>128	0.016	>128	2	I
AT4	0.016	>128	0.016	>128	2	I
AN1	0.03	>128	0.03–0.06	>128	2–3	I

OLO, olorofim; FLU, fluconazole; I, indifference.

### Terbinafine shows indifference with olorofim

The response to combinations of terbinafine and olorofim varied between *Aspergillus* species (Table 6 and Figure 1). Terbinafine increased olorofim MICs by one dilution in the *A. fumigatus* isolates but reduced olorofim MICs 8- to 16-fold in the non-*fumigatus* species. Terbinafine MICs did not change in combination for six of the eight strains; only the two azole-resistant *A. fumigatus* showed a one dilution increase in terbinafine MIC. Regardless of these changes in olorofim susceptibility, FICI calculation revealed indifference between the two agents.

**Table 6.** Interaction between olorofim and terbinafine in *Aspergillus* spp.

	Agent alone (mg/L)		Combination (mg/L)			
Strain	OLO	TER	OLO	TER	FICI	Interaction
AF293	0.016	1	0.03	2	4	I
NIH4215	0.03	4	0.06	4	3	I
ATCC204305	0.016–0.03	2	0.03	2	2–3	I
F17294	0.016–0.03	1	0.03–0.06	2	4	I
F11628	0.016	2	0.03	1–2	2.5–3	I
ATCC204304	0.016	≤0.5	≤0.002	≤0.5	1.125	I
AT4	0.016	≤0.5	≤0.002	≤0.5	1.125	I
AN1	0.03	0.5	0.002	0.5	1.0625	I

OLO, olorofim; TER, terbinafine; I, indifference.

### Amphotericin B and olorofim behave indifferently against A. fumigatus, A. flavus and A. terreus but olorofim antagonizes the effect of amphotericin B against A. niger

In combination, amphotericin B and olorofim were indifferent in seven of the eight *Aspergillus* isolates tested (Table 7 and Figure 1). Combination MICs for the three azole-susceptible *A. fumigatus* isolates were reduced by one to two dilutions for both agents compared to each drug alone. The two azole-resistant *A. fumigatus* isolates and the *A. flavus* isolate showed no change in olorofim or amphotericin B MICs in combination. By contrast, combination MICs were lower than each agent alone for *A. terreus*; the olorofim MIC was reduced by one dilution while the amphotericin B MIC was reduced by four dilutions. However, all FICI calculations reflected indifference between the two agents for these species. Surprisingly, olorofim was observed to antagonize amphotericin B activity in the *A. niger* isolate, increasing the polyene MIC by three dilutions while the olorofim MIC was unchanged.

**Table 7.** Interaction between olorofim and amphotericin B in *Aspergillus* spp.

	Agent alone (mg/L)		Combination (mg/L)			
Strain	OLO	AMP	OLO	AMP	FICI	Interaction
AF293	0.016–0.03	2	0.008–0.03	1–2	1–2	I
NIH4215	0.016–0.03	1	0.008	1	1.25–1.5	I
ATCC204305	0.016	1	0.008–0.016	0.5–1	1–2	I
F17294	0.016	0.5–1	0.016	0.5–1	2	I
F11628	0.016	0.5	0.016	0.5	2	I
ATCC204304	0.016	2	0.016	2	2	I
AT4	0.016	4	0.008	0.25	0.5625	I
AN1	0.03	0.25	0.03	2	9	A

OLO, olorofim; AMP, amphotericin B; A, antagonism; I, indifference.

### Caspofungin and olorofim show indifference

Caspofungin had no effect on olorofim MICs in six of the eight isolates tested (Table 8 and Figure 1). However, olorofim MICs were reduced by up to three dilutions and caspofungin MECs were reduced by one dilution in *A. flavus* and *A. terreus* (ATCC204304 and AT4, respectively) in combination. Regardless of these endpoint changes, FICI calculation revealed indifference between caspofungin and olorofim in all strains.

**Table 8.** Interaction between olorofim and caspofungin in *Aspergillus* spp.

	Agent alone (mg/L)		Combination (mg/L)			
Strain	OLO	CAS	OLO	CAS	FICI	Interaction
AF293	0.008	0.125–0.25	0.008	0.125–0.25	2	I
NIH4215	0.016	0.25	0.016	0.25	2	I
ATCC204305	0.016	0.125	0.016	0.125	2	I
F17294	0.016	0.125	0.016	0.125	2	I
F11628	0.016	0.125	0.016	0.125	2	I
ATCC204304	0.016	0.5	0.002–0.004	0.25	0.625–0.75	I
AT4	0.008	0.125	0.002–0.008	0.06–0.125	0.75–2	I
AN1	0.016	0.25	0.016	0.25	2	I

OLO, olorofim; CAS, caspofungin; I, indifference.

### Olorofim shows indifference with fluconazole or voriconazole in Candida species

Although *Candida* species are intrinsically resistant to olorofim, it is possible it may be given to patients receiving azole prophylaxis or treatment for *Candida* infections. The effect of olorofim on the *in vitro* activity of fluconazole (Table 9 and Figure 3) and voriconazole (Table 10) was investigated against *C. parapsilosis*, *C. albicans* and *C. glabrata.* As expected, olorofim MICs were above the highest concentration tested (>8 mg/L) for all three species. Fluconazole MICs were not affected by olorofim for *C. parapsilosis* and *C. glabrata* but increased by one dilution for *C. albicans*. FICI calculations for all three species revealed indifference between fluconazole and olorofim at the concentrations tested. The voriconazole MIC was not altered by olorofim for *C. glabrata* but increased by one dilution in both C. *parapsilosis* and *C. albicans*. As was the case for fluconazole, FICI values confirmed voriconazole and olorofim were indifferent in combination.

**Table 9.** Interaction between fluconazole and olorofim in *Candida* spp.

	Agent alone (mg/L)		Combination (mg/L)			
Strain	FLU	OLO	FLU	OLO	FICI	Interaction
ATCC22019	2	>8	2	>8	2	I
CA	0.25	>8	0.5	>8	3	I
CG	8	>8	8	>8	2	I

FLU, fluconazole; OLO, olorofim; I, indifference.

**Table 10.** Interaction between voriconazole and olorofim in *Candida* spp.

	Agent alone (mg/L)		Combination (mg/L)			
Strain	VOR	OLO	VOR	OLO	FICI	Interaction
ATCC22019	0.03	>8	0.06	>8	3	I
CA	0.004	>8	0.008	>8	3	I
CG	0.5	>8	0.5	>8	2	I

VOR, voriconazole; OLO, olorofim; I, indifference.

## Discussion

Previous data on the interaction between olorofim and azoles *in vitro* are varied; antagonism between voriconazole and olorofim was observed against a wild-type *A. fumigatus* isolate but not against an azole-resistant isolate,^26^ nor for itraconazole and olorofim against *M. mycetomatis*.^29^ Furthermore, outcomes were similar for monotherapy and for olorofim in combination with various azoles in a Phase 2 study of olorofim.^30^ The current study investigated the interactions between olorofim and various antifungals against a panel of eight *Aspergillus* isolates *in vitro* using standardized EUCAST methodology in checkerboard assays. Furthermore, interactions between olorofim and fluconazole or voriconazole were assessed in three *Candida* strains.

In this study, a clear pattern of unidirectional antagonism of olorofim activity was seen only with the mould-active azoles: azole MIC values were unaffected or increased by one dilution but the olorofim MIC increased up to 32-fold (Figure 1). The most pronounced effect was observed with posaconazole or isavuconazole, where unequivocal antagonism was seen with all strains. For voriconazole, antagonism was determined for five of the eight isolates with indifference seen for the remaining three isolates. Antagonism of olorofim activity was observed with both azole-susceptible and azole-resistant isolates and was most prominent at or adjacent to the azole MIC. It is interesting to note that the L98H/TR34 *A. fumigatus* mutant behaved similarly in this study as previously observed in that voriconazole and olorofim behaved indifferently, but for the G138C azole-resistant mutant, the FICI reflected antagonism.^26^

**Figure 2.**
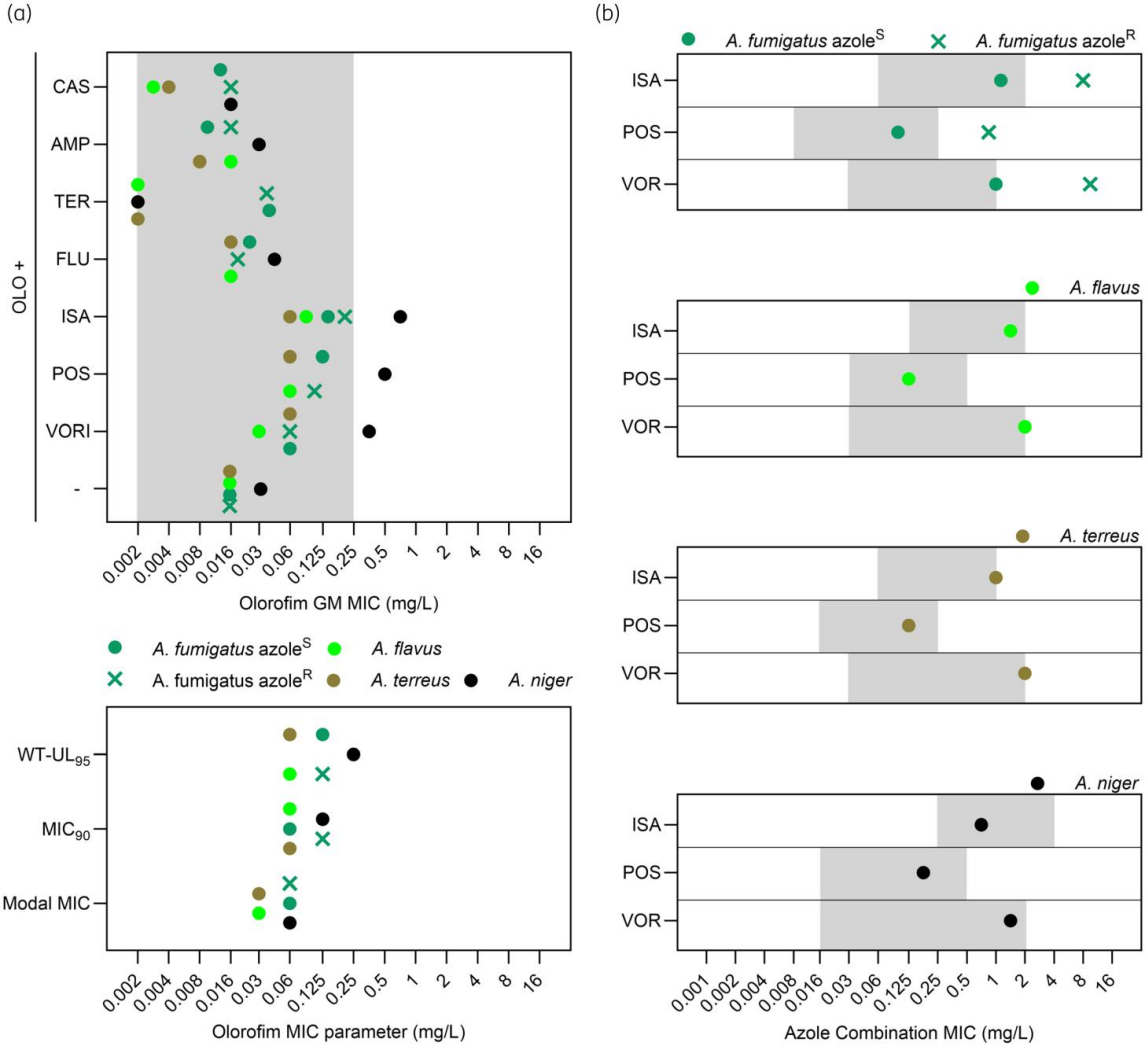
Combination MICs remain within wild-type distributions for *Aspergillus* spp., other than for *A. niger.* (a) Top—GM olorofim MIC values alone (−) or in combination with the other antifungal agents tested. Grey box represents wild-type *Aspergillus* spp. MIC distribution.^22^ Bottom, published distribution parameters are included for comparison.^22^ (b) GM azole MIC values in combination with olorofim. Grey boxes show the wild-type distribution of MICs seen for each agent, from the lowest observed MIC up to the ECOFF value.^37,39,43,44^ OLO, olorofim; VOR, voriconazole; POS, posaconazole; ISA, isavuconazole; TER, terbinafine; AMP, amphotericin B; CAS, caspofungin.

Importantly, the magnitude of the changes in MIC for both the azoles and olorofim is limited. For olorofim, the increase in MIC was typically two to four dilutions and the resulting MIC was ≤0.25 mg/L for all isolates other than the single tested isolate of *A. niger* where olorofim MICs of 0.25–1 mg/L were noted in combination testing with the three mould-active azoles (Figure 1). Interpretive breakpoints for olorofim have not yet been established but the wild-type range for *Aspergillus* spp. extends to 0.25 mg/L.^21,22^ The wild-type range for *Lomentospora prolificans* and *Scedosporium* similarly extends to 0.25–0.5 mg/L^15,41^ and importantly *in vivo* murine infection models show efficacy with isolates from all three genera at similar doses.^13,42^ We note that most of the increased olorofim combination MICs fall within the wild-type olorofim MIC distribution for *Aspergillus* spp. and within various observed population parameters (Figure 2a).^22^ As such, these raised MICs are wild-type in the context of published MIC ranges for olorofim, in contrast to raised MICs caused by DHODH mutations (>8 mg/L).^23^ Further studies are required to establish the upper limit of *in vitro* susceptibility of *Aspergillus* isolates that exhibit successful clinical response. In contrast, azole MICs were largely unchanged within wild-type MIC distributions for sensitive isolates and below the epidemiological cut-off (ECOFF) values (Figure 2b).^39,43–45^ Unsurprisingly, azole-resistant isolates maintained high azole MICs in combination but were still responsive to olorofim, a result reflected *in vivo*.^31^

The mechanism underlying the antagonism between and olorofim and the mould-active azoles remains to be fully elucidated as studies thus far have only investigated cellular responses to short-term exposure (1–4 hours) to itraconazole or olorofim, individually.^26^ Itraconazole exposure was seen to upregulate multiple genes in the pyrimidine biosynthetic pathway while olorofim exposure only upregulated one. Pathways that produce pyrimidine precursors were upregulated by both agents. Thus, it can be inferred that the combination of these antifungals may lead to dual upregulation of pyrimidine biosynthesis and as such may cause the slightly increased resilience to olorofim *in vitro*. Disconnecting the transcriptional crosstalk between azoles and the pyrimidine biosynthesis pathway by replacing the promoters of the genes *pyrABCN* and *pyrD* decreased the antagonism observed, supporting this as a mechanism contributing to the observed MIC changes.^26^ In line with this hypothesis, antagonism of olorofim activity was seen regardless of azole susceptibility and was strongest at the highest survivable concentration of the azole, indicating that the degree of antagonism is determined by azole concentration mediated through Cyp51A. Different azoles may vary in their impact on transcription upregulation, which may account for variations in the degree of antagonism seen with olorofim and voriconazole compared with posaconazole and isavuconazole. Other mechanisms may also contribute to antagonism, such as the upregulation of drug-efflux transporters including Mdr1, AbcA and Cdr1B and the activation of multiple stress responses observed following azole treatment of *A. fumigatus*.^46^ Terbinafine also affects the ergosterol synthesis pathway but showed indifference with olorofim (Figure 1). Olorofim and terbinafine have been used in combination to successfully treat refractory *Microascus* infections.^9^ The contrast in olorofim interaction between the azoles and terbinafine may be due to both their targeting the ergosterol synthesis pathway at different steps and differential transcriptional responses. The reduction of olorofim MICs in the presence of terbinafine for *A. flavus* and *A. terreus* is interesting and warrants further study from a mechanistic viewpoint and may provide clinical insight for treatment of rare mould infections where terbinafine is used in combination therapy.^47^

While there is antagonism observed *in vitro* between olorofim and mould-active azoles against *Aspergillus* spp., the consequences in clinical practice are uncertain. However, Phase II clinical data showed that in a cohort of 51 aspergillosis patients, treatment with either olorofim and a concomitant azole (*N *= 16) or olorofim monotherapy (*N* = 37) had no significant difference in all-cause mortality and overall response at two different timepoints.^24,30^ In addition, our results are in line with *in vivo* findings (Darlow *et al.*, 2025, unpublished results) that a combination of olorofim and posaconazole suppresses azole-susceptible infection better than each agent alone.^31^ Logically, clinical response to olorofim negates the need for a mould-active azole when treating an olorofim-susceptible organism, therefore the clinical reality of mould being exposed to mould-active azoles and olorofim together may be transient during a switch from one drug to the other.

**Figure 3.**
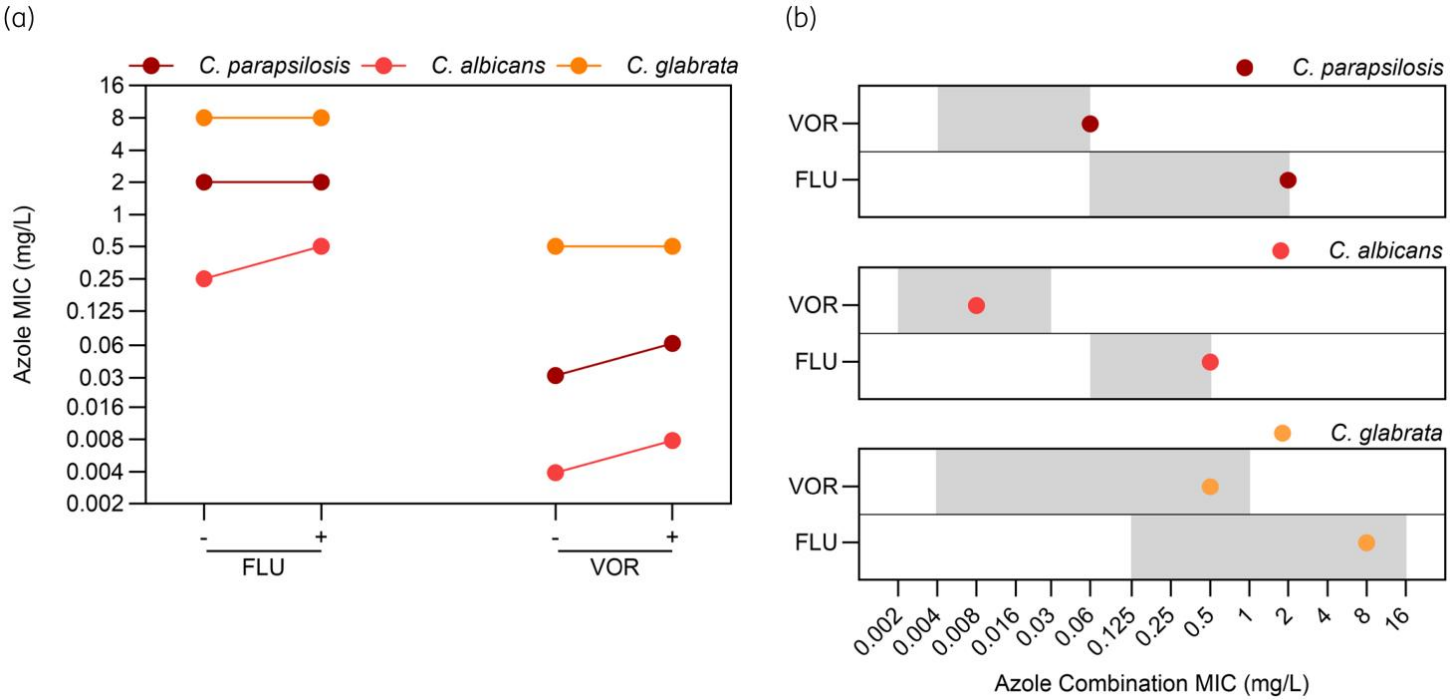
Combination azole MICs remain within wild-type distributions for all *Candida* spp. (a) GM MIC values for the azoles tested in the absence (−) or presence (+) of olorofim. (b) GM azole MIC values in combination with olorofim. Grey boxes show the wild-type distribution of MICs seen for each agent, from the lowest observed MIC up to the ECOFF value.^37–39^ VOR, voriconazole; FLU, fluconazole.

In contrast to the mould-active azoles, no antagonism was observed between olorofim and fluconazole in *Aspergillus* even at high fluconazole concentrations (Figure 1). Though olorofim increased the fluconazole MIC by one dilution in *C. albicans*, no interaction was observed between the two agents for all *Candida* species tested; this outcome was also observed for voriconazole (Figure 3a). Combination MICs for these azoles against *Candida* spp. remained within the WT ECOFF range for each species (Figure 3b).^37–39^ These data suggest that combined dosing of olorofim with fluconazole may be possible and could allow treatment/prophylaxis of mould and yeast simultaneously.

**Figure 4.**
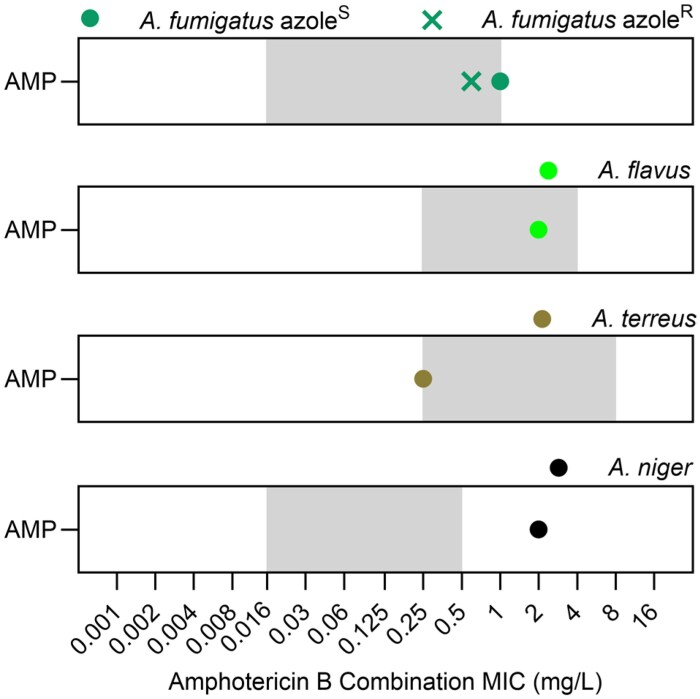
Combination amphotericin B MICs remain within wild-type distributions for all *Aspergillus* spp. except *A. niger*. GM amphotericin B MIC values in combination with olorofim. Grey boxes show the wild-type distribution of MICs seen for amphotericin B, from the lowest observed MIC up to the ECOFF value.^37,48^ AMP, amphotericin B.

No interactions were observed between amphotericin B and olorofim except for the *A. niger* isolate tested, where olorofim increased the amphotericin B MIC 8-fold to 2 mg/L, one concentration above the tentative ECOFF value *for A. niger* (Figures 1 and 4).^37,48^ Olorofim and amphotericin B combination therapy may not be the optimal clinical approach for *A. niger*; further experiments are required to see if this effect can be generalized to *A. niger* and related section species and whether this phenomenon extends to *in vivo* settings.

Overall, while this study, using a limited number of strains in a static assay, found that the olorofim MIC is affected by some antifungal agents, MICs for both agents remain relatively low. There are several intriguing findings in this study which require further investigation. The strongest degree of antagonism with azoles or amphotericin B and olorofim occurred with *A. niger*, reasons for this are unclear but warrant further work with this species. In addition, *in vitro* studies of antifungal combinations should be extended to non-*Aspergillus* moulds within the spectrum of olorofim activity.
